# Progress in Medicine

**Published:** 1899-10-21

**Authors:** 


### Progress in Medicine.

### DISEASES OF THE KIDNEY.

(*Continued from page* 32.)

**Urology.**—Essential renal hæmaturia is discussed by Debairieux,[26] who reports a case in a young woman. The disease had lasted six months, following some febrile affection, and was accompanied by pain, sometimes like that of severe renal colic. The urine was normal, except for some blood and the corresponding amount of albumen. A nephrotomy was performed, but neither stone nor new growth was found, and the patient has remained completely well ever since. Many other cases are referred to in which no adequate lesion was found. The pathology is entirely unknown, and no remedies appear to have any effect, though the hæmorrhage may disappear spontaneously or after an operation. H. T. Bewley[27] gives an account of a case of chyluria which occurred in a woman who had been in the tropics, but in whom no filiariæ could be discovered. The urine was sometimes as white as milk, and a layer of cream would rise to the top after standing. There were no tube casts, cells, or sugar, but a normal amount of urea. Under rest in bed, with strychnia and perchloride of iron, the condition passed away almost entirely. It is suggested that some communication between the lymphatics and the urinary passages existed, possibly from the obstruction caused by filiariæ which had since died. This view is generally accepted, though other explanations have been put forward.

Péhu[28] discusses the value of tube casts in the diagnosis of renal disorders. He divides them into those produced (*a*) by transudation, (*b*) by desquamation, and (*c*) by proliferation of striated epithelial cells. Granular casts are indicative of nephritis affecting the tubules of the cortex, and are of more importance than other forms. In the stage of sclerosis colloid casts may accompany the granular ones. Alkaptonuria was the subject of a paper by A. E. Garrod.[29] The fluid rapidly becomes dark, especially if an alkali be added. It reduces Fehling's solution when heated with it. Only 31 instances have been recorded, and generally it lasts for life without injury to the patient. The writer found reason to accept the view that the cause of the phenomenon was homogentisinic acid, which might be due to some perverted metabolism. Its presence might lead to a mistaken diagnosis of sugar. Oxaluria may be accompanied with general nerve symptoms; but, according to R. F. Williams,[30] the latter are not necessarily caused by the oxaluria. Symptoms of Bright's disease may also be produced by the causes which lead to the oxaluria, and finally the latter by the irritation it causes may actually set up Bright's disease. R. R. Kine, in the "Postgraduate," recommends in oxaluria treatment of indigestion, diuretics, gymnastics to increase oxidation, and peroxide of hydrogen in hot water early every morning. E. G. Klein,[31] in an examination of the urine of 250 insane persons, found oxalates in six of his depressed patients, and notices the recovery from melancholia and oxaluria together in some cases as though they had a common origin.

**Albumosuria.**—Bradshaw and Warrington[32] reported a case in which this substance was noted during life, with fragility of the ribs, sternum, and vertebræ. After death well marked multiple myeloma was found in the bones. Six other cases with bone disease were on record, and it appears that the affection is quite distinct from osteomalacia. Kelynack[33] quotes a case of Rosin's, where albumose was found together with sarcomatous growths in the ribs. Kahlu[34] first suggested in 1889 some relation between albumosuria and bone disease. The lesion consists in a cellular growth which proliferates in the cancellous tissue and absorbs the hard parts, so that the bone is reduced to a mere shell. The albumose is found in large quantities and for a long period, and differs from both albumen and the digestive albumoses.

**Albuminuria.**—S. West[35] comments on the difficulty of distinguishing nucleo albumen from serum albumen and globulin. He finds that the former is precipitated more quickly by acetic acid, and on dilution the cloud is not diminished. With Heller's test it gives a ring rather above the line of junction, or a general haze, instead of a ring at the junction of the urine and acid. It gives no reaction to ferro-cyanide of potash and acetic acid unless it be present in quantity. True mucus also gives no reaction with this test if the two reagents are first mixed and the urine added after. A saturated solution of citric acid easily precipitates nucleo-albumen, but affects the serum form with difficulty. Lolke[36] separates albumoses from peptones by adding carefully a dilute solution of perchloride of iron. The albumoses will be precipitated if the reagent is not used in excess, which would redissolve them. Purdy[37] recommends for the quantitative estimation of albumen that 10 cc. of urine should be precipitated with ferro-cyanide (3 cc. of a 1 in 10 solution) and 2 cc. of a 50 per cent. acetic acid. After 10 minutes standing the tubes are centrifugalised and the amount read off by the aid of a table. With special tubes the amount can be measured within 1 part in 5,000. G. Rosenfeld,[38] however, shows that Esbach's tubes gave estimates which did not vary from analyses by weighing (Scherer's method) more than 1 part in 2,500 for the series of cases tested.

O. C. Ludlow[39] warmly attacks Truax's test—alcohol —as fallacious. He found that the results were entirely opposed to those given by known agents, and a reaction seemed most common in urines of high colour and specific gravity. In 50 per cent. of his cases where the reaction was obtained, other tests showed the absence of albumen. He has not been able to discover what are the substances which cause a precipitate on adding strong alcohol to urine, but they do not include serum albumen. He prefers on the whole the nitro-magnesian test, performed by adding one part of nitric acid to five of a saturated solution of magnesium sulphate, which gives a ring more clearly than pure nitric acid does. P. T. Cammidge,[40] in a useful criticism of the ordinary tests, also points out that the ring formed by nucleo-albumen with cold nitric acid is different from that given by serum albumen, being higher up, and is equally strong if the urine is diluted, while that due to the serum form becomes fainter. The best and most convenient test is salicyl-sulphonic acid. It precipitates albumens and albumoses alone, and the latter disappear on heating. It is, however, more delicate than necessary for clinical work, and precipitates nucleo-albumen. To correct it he employs cold nitric acid, which is less delicate, and enables us to distinguish the nucleo form as shown above.

For the separation of the urine from the two kidneys without the trouble of catheterising the ureters, M. L. Harris[41] has devised a segregator which pushes up the bladder in the middle line and enables that which flows from either ureter to be drawn off separately. He describes its use minutely in a recent article, and warns us that besides estimating the state of the urine from a diseased kidney we should also measure the "functional capacity" of the more healthy one before determining upon nephrectomy. Thus we should determine the amount of urine per kilo of the body-weight excreted per minute and the urea contained in it. Thus there should be 2 per cent. of urea and ·008 cc. of urine per kilo excreted in a minute by each healthy kidney. If this rule is not observed removal of a diseased kidney may leave the body with no means of carrying on elimination. Urotropin is highly praised by Kelly[42] in cystitis and phosphaturia. He gives in bad cases as much as 20 grains twice a day, and if the urine is very alkaline adds a little dilute mineral acid. He has by its continued use rendered urine permanently clear after the patient had passed pus for fifteen years. The ordinary dose is 7 grains.

[26] Clin. Jour., Feb. 22. [27] Dublin Jour. Med. Sc., Feb. [28] Med. Rec., May 27. [29] Brit. Med. Jour., May 13. [30] New York Med. Jour., May 20. [31] New York Med. Jour., Mar. 18. [32] Lancet, Mar. 18. [33] Practitioner, Mar. [34] Brit, Med. Jour., Mar. [35] Brit. Med. Jour., Feb. 25. [36] Les Nouveaux Remèdh., Feb. [37] Med. Rec., June 17. [38] Berlin Klin. Woch., 1898, No. 30. [39] Med. Rec., Jan. 7. [40] Lancet, April 22. [41] Med. Rec., April 1. [42] Med. Censor, May.

